# Identification of microRNA profile specific to cancer stem-like cells directly isolated from human larynx cancer specimens

**DOI:** 10.1186/s12885-016-2863-3

**Published:** 2016-11-05

**Authors:** Omer Faruk Karatas, Ilknur Suer, Betul Yuceturk, Mehmet Yilmaz, Buge Oz, Gulgun Guven, Harun Cansiz, Chad J. Creighton, Michael Ittmann, Mustafa Ozen

**Affiliations:** 1 Molecular Biology and Genetics Department, Erzurum Technical University, Erzurum, Turkey; 2 Department of Medical Genetics, Istanbul University Cerrahpasa Medical School, Istanbul, Turkey; 3 Advanced Genomics and Bioinformatics Research Center, The Scientific and Technological Research Council of Turkey (TUBITAK), Gebze, Kocaeli Turkey; 4 Department of Otorhinolaryngology, Cerrahpasa Medical School, Istanbul University, Istanbul, Turkey; 5 Department of Pathology, Cerrahpasa Medical School, Istanbul University, Istanbul, Turkey; 6 Department of Medicine and Dan L. Duncan Cancer Center Division of Biostatistics, Baylor College of Medicine, Houston, TX USA; 7 Department of Pathology & Immunology, Baylor College of Medicine, Houston, TX 77030 USA; 8 Michael E. DeBakey VAMC, Houston, TX 77030 USA

**Keywords:** Cancer stem-like cells, MicroRNAs, Larynx cancer, CD133, microRNA-signature

## Abstract

**Background:**

Emerging evidences proposed that microRNAs are associated with regulation of distinct physio-pathological processes including development of normal stem cells and carcinogenesis. In this study we aimed to investigate microRNA profile of cancer stem-like cells (CSLCs) isolated form freshly resected larynx cancer (LCa) tissue samples.

**Methods:**

CD133 positive (CD133^+^) stem-like cells were isolated from freshly resected LCa tumor specimens. MicroRNA profile of 12 pair of CD133^+^ and CD133^−^ cells was determined using microRNA microarray and differential expressions of selvected microRNAs were validated by quantitative real time PCR (qRT-PCR).

**Results:**

MicroRNA profiling of CD133^+^ and CD133^−^ LCa samples with microarray revealed that miR-26b, miR-203, miR-200c, and miR-363-3p were significantly downregulated and miR-1825 was upregulated in CD133^+^ larynx CSLCs. qRT-PCR analysis in a total of 25 CD133^+^/CD133^−^ sample pairs confirmed the altered expressions of these five microRNAs. Expressions of miR-26b, miR-200c, and miR-203 were significantly correlated with miR-363-3p, miR-203, and miR-363-3p expressions, respectively. Furthermore, *in silico* analysis revealed that these microRNAs target both cancer and stem-cell associated signaling pathways.

**Conclusions:**

Our results showed that certain microRNAs in CD133^+^ cells could be used as cancer stem cell markers. Based on these results, we propose that this panel of microRNAs might carry crucial roles in LCa pathogenesis through regulating stem cell properties of tumor cells.

**Electronic supplementary material:**

The online version of this article (doi:10.1186/s12885-016-2863-3) contains supplementary material, which is available to authorized users.

## Background

Larynx Cancer (LCa) is an aggressive neoplasm constituting approximately 1 to 2.5 % of all human cancer cases worldwide [1–3]. It is known as one of the most common tumor types of the head and neck region [4]. Despite notable enhancements in the therapeutic options; treatment outcome, prognosis, and 5-year survival rates for LCa remained almost unchanged in nearly past two decades [5, 6]. Therefore, more studies exploring the underlying mechanisms of LCa pathogenesis are urgently needed for better understanding of LCa development and providing more effective treatment strategies.

Emerging evidences propose the idea that a highly malignant rare subpopulation of tumor cells exhibits stem cell-like features [7]. This reservoir of stem-like cells within the bulk tumor is considered as tumor-initiating or cancer stem-like cells (CSLCs) with their unique capacity for unlimited self-renewal, multi-lineage differentiation, and ability for initiation, maintenance, and spread of tumor [8]. CSLCs has been proven to be present in a variety of tumors including lung, brain, breast, prostate, colon, ovarian, and head and neck cancers [9, 10] and are considered as the driving force for tumor relapse, metastasis, and chemo-radioresistance [11–13].

We recently demonstrated that stem-like cells are highly enriched in CD133 overexpressing LCa cells, which are profoundly positive for stem cell markers including SOX2, OCT4, KLF4 and ABCG2 [14]. Furthermore, several studies have pointed to certain gene expression signatures specific to embryonic stem cells in acquisition and maintenance of the biological features of CSLCs [15–17]; however, the underlying mechanisms are not yet completely understood. Therefore, elucidation of genetic and epigenetic circuits regulating the stem cell characteristics of CSLCs might help understanding the molecular basis of carcinogenesis.

There is an increasing body of evidence demonstrating that microRNAs (miRNAs) are associated with regulation of distinct physio-pathological processes including development of normal stem cells and carcinogenesis [18, 19]. MiRNAs are 21–25 nucleotides long, endogenously synthesized, noncoding RNAs that are involved in post-transcriptional gene silencing of target messenger RNAs (mRNAs) through binding 3′-untranslated regions (3′UTR) [20]. Deregulation of miRNAs has been linked to several diseases including cancer, where they can act as oncogenes or tumor suppressors. Recent studies implied miRNAs as crucial molecular players in cancer initiation, progression, and metastasis [21–23].

Recently, utilization of Dicer or Dgcr8 knockout mice, lacking global miRNA processing capability, demonstrated that cells failed in self-renewal since stem cell specific markers couldn’t be downregulated. This indicated the significance of miRNAs in establishing stem cell identity [24, 25]. Besides, several miRNAs have been proposed to have direct roles in survival of CSLCs [8, 26, 27]. Therefore, understanding the contribution of miRNAs in acquisition and maintenance of CSLCs will provide the opportunity to develop miRNA-based therapeutic tools [28].

In this study, we investigated genome-wide miRNA expression profile of laryngeal CSLCs enriched for CD133 surface marker to identify a CSLCs specific miRNA signature.

## Methods

### Patients

This study has been reviewed and approved by an institutional review board of Istanbul University, Cerrahpasa Medical School (IRB No: 35697). 25 LCa tumor tissue specimens were obtained from Department of Otorhinolaryngology, Cerrahpasa Medical School, Istanbul University. None of the patients received radiotherapy, chemotherapy or immunotherapy subsequent to the surgery. The characteristics of the patients including age, gender, T classification and histological grade were summarized in Table 1. Freshly resected tumor tissues were collected immediately after the surgery and processed for CSLCs isolation. Patients were included into the study upon giving their written informed consents. We also obtained consent to publish from the participants.

**Table 1:** Clinico-pathological information of the patients

	LCa Subjects
Age	
≤ 60	18
> 60	7
Gender	
Male	23
Female	2
T Classification	
T1 and T2	4
T3 and T4	21
Histological grade	
II	9
III	16

### Cancer stem cell isolation

CD133 positive (CD133^+^) cells were isolated from freshly resected and physically/enzymatically dissociated tumor tissue samples using Magnetic-activated Cell Sorting (MACS) technique (Miltenyi Biotech, Bergisch Gladbach, Germany) and “EasySep Positive Selection Human PE Selection Kit (StemCell Technologies, (Vancouver, BC, Canada)” following the manufacturer’s protocol. Shortly, fresh tumor tissue samples were physically minced with a scalpel and exposed to enzymatic dissociation using 400 μg/ml Collagenase enzyme (GIBCO, New York, USA) at 37 °C for 3 h. Dissociated cells were filtered using a 70-μm cell strainer to get a single cell suspension. Cells were labeled with CD133/2-PE (Miltenyi Biotech clone AC133) antibody. After magnetic sorting, CD133 enriched (CD133^+^) and remaining (CD133^−^) cell populations from the same tissue samples were immediately washed and homogenized in “Lysis/Binding Buffer” of “mirVana miRNA Isolation Kit” (Ambion, Darmstadt, Germany) for further RNA isolation.

### RNA isolation

Total RNA was isolated from CD133^+^ and CD133^−^ cells collected from LCa tumor samples using “mirVana miRNA Isolation Kit” (Ambion, Darmstadt, Germany) following the manufacturer’s instructions. The purities and concentrations of RNA samples were determined spectrophotometrically using NanoDrop ND-2000c (Thermo Fisher Scientific, Inc., Wilmington, DE).

### MiRNA microarray and data analysis

Genome wide microRNA profiling of 12 pairs of CD133^+^and CD133^−^ cell populations collected from 12 tumor samples were performed using Agilent Human miRNA Microarray (V19). 100 ng of total RNA from each sample were labeled with Cy3 by using Agilent miRNA labeling kit following manufacturer’s instructions. Labeled RNAs were heat denatured and hybridized to Agilent 8x15k miRNA microarray V19 comprised of 2006 miRNAs from Sanger miRBase (release 19) at 55 ° C for 20 h. After hybridization, slides were immediately washed and scanned in Agilent Microarray Scanner with Surescan High Resolution Technology (Agilent Technologies, Santa Clara, CA). Feature Extraction v10.7.3.1 (Agilent Technologies, CA) software was used to extract all features of the data obtained from the scanned images. Data were normalized by quantile normalization, using Bioconductor 2.10 with R version 2.15. Tumor samples were profiled on one of two different Agilent grid designs: Agilent-031181 (four pairs of CD133^+^ and CD133^−^ cell populations collected from four tumor tissue samples) and Agilent-053955 (eight pairs of CD133^+^ and CD133^−^ cell populations collected from eight tumor tissue samples); to correct for inter-platform differences, values were averaged by probe set, and each patient profile was compared with its corresponding CD133^−^ profile by paired analysis (both pairs being represented on the same platform). *P* values and fold changes were calculated for each feature, using log-transformed values and paired t-test by patient. Differentially expressed miRNAs with *P* < 0.01 and 1.4-fold change were selected for further confirmation by RT-PCR. Array data have been deposited into the Gene Expression Omnibus (accession GSE69128).

### MiRNA cDNA synthesis and quantitative reverse-transcription PCR

For the miRNA selection after microarray analysis, significantly deregulated miRNA probes were listed according to their fold changes. Then, top 10 upregulated and downregulated probes were selected for further literature search. We investigated the following properties and statuses for every single microRNA; deregulation in cancer, deregulation in larynx cancer, deregulation in head and neck cancers, expression in stem cells, and functional studies in stem cells. For top 10 upregulated microRNAs (hsa-miR-197-3p, hsa-miR-574-3p, hsa-miR-885-5p, hsa-miR-483-3p, hsa-miR-1281, hsa-miR-328, hsa-miR-4254, hsa-miR-4290, hsa-miR-1825, hsa-miR-766-3p), we included those have been shown to be deregulated in cancer, and have either expression data or functional studies in stem cells. Only hsa-miR-574-3p, hsa-miR-328, and hsa-miR-1825 met these criteria. For top 10 downregulated microRNAs (hsa-miR-106b-5p, hsa-miR-26b-5p, hsa-miR-494, hsa-miR-425-5p, hsa-miR-363-3p, hsa-miR-15b-5p, hsa-miR-185-5p, hsa-miR-150-5p, hsa-miR-223-3p, hsa-miR-142-5p), we included those have been shown to be deregulated in cancer (having no controversial expression status; some of these microRNAs have been shown to be upregulated in some cancer types, whereas, downregulated in other cancer types), and have either expression data or functional studies in stem cells. Only hsa-miR-26b-5p, hsa-miR-363-3p, and hsa-miR-223-3p met these criteria. Besides, we included miR-200c and miR-203 since they are strongly associated with stemness and cancer, although these miRNAs are not in the top 10 differentially expressed miRNAs.

To validate the differential expression of miR-26b, miR-200c, miR-203, miR-223, miR-328, miR-363-3p, 574-3p, and miR-1825, a total of 25 pairs of CD133^+^ and CD133^−^ cell populations collected from 25 tumor samples including those used in microarray experiments were studied. First strand DNA (cDNA) synthesis was carried out with 30 ng of total RNA from each sample using miRNA specific primers purchased from Applied Biosystems and “TaqMan MicroRNA Reverse Transcription Kit” according to the manufacturer’s protocol (Applied Biosystems, Foster City, CA). MiRNA expression analysis by quantitative reverse-transcription PCR was carried out using a Roche LightCycler480-II real-time thermal cycler (Roche, Switzerland). TaqMan Universal Master Mix and TaqMan amplification kits (Applied Biosystems, Foster City, CA) were used. Expression levels of miRNAs in each CD133^+^ cell population were calculated as compared to CD133^−^cells collected from the same tumor tissue sample. Therefore, expression levels of CD133^−^ cells were fixed to 1 for every sample. RNU43 was used for normalization of miRNA expression analyses. Each experiment was performed in duplicate. The relative quantification analysis was performed by delta-delta-Ct method as described previously [29].

### Statistical analysis

Data were plotted as mean ± standard error of the mean. Statistical analysis was carried out using two-sided Student’s t-test. Pearson Correlation test was used to show the correlation of differentially expressed mRNAs. A *p*-value < 0.05 was considered as statistically significant. MiRWalk 2.0 [30] and miRTarBase [31] *in silico* tools were used to estimate the predicted miRNA targets and to evaluate the validated miRNA targets, respectively. MiRWalk 2.0 is a freely accessible archive of predicted and experimentally verified miRNA-target interactions [30], whereas miRTarBase is a miRNA-target interactions database, where the collected miRNA-target interactions are validated experimentally by reporter assay, western blot, microarray and next-generation sequencing experiments [31]. In both tools, miRBase IDs were used as inputs. MiRWalk 2.0 and miRTarBase provide the gene list of predicted/validated targets of miRNAs and predicted gene interactors of miRNAs based on functional assays, respectively. The number of tumor suppressor and oncogenic targets of miRNAs were determined using miRWalk 2.0 tool. While predicting the targets of miRNAs, in ‘Step 4: Enriched functional patterns’, oncogene or tumor suppressor was selected as gene class, and the results provided the number of tumor suppressor and oncogenic targets of the specified miRNA. String [32] tool was utilized to prepare schematic representation of miRTarBase results. DIANA-miRPath was used for miRNA pathway analysis web-server [33].

## Results

### Subject characteristics

Twenty five LCa patients were included in this study to explore the miRNA expression status of CD133^+^ larynx CSLCs and remaining CD133^−^ non-CSLCs. The diagnosis of patients has been confirmed histopathologically in Istanbul University, Cerrahpasa Medical School. All patients except one were men and their ages ranged from 44 to 84 years (median, 64 years). Histological grades of tumor specimens were determined according to World Health Organization classification, which demonstrated that 9 tumors were grade II and 16 tumors were grade III. Clinical characteristics of the patients are summarized in Table 1.

### MiRNA profile of CD133^+^ larynx CSLCs

**Fig. 1:**
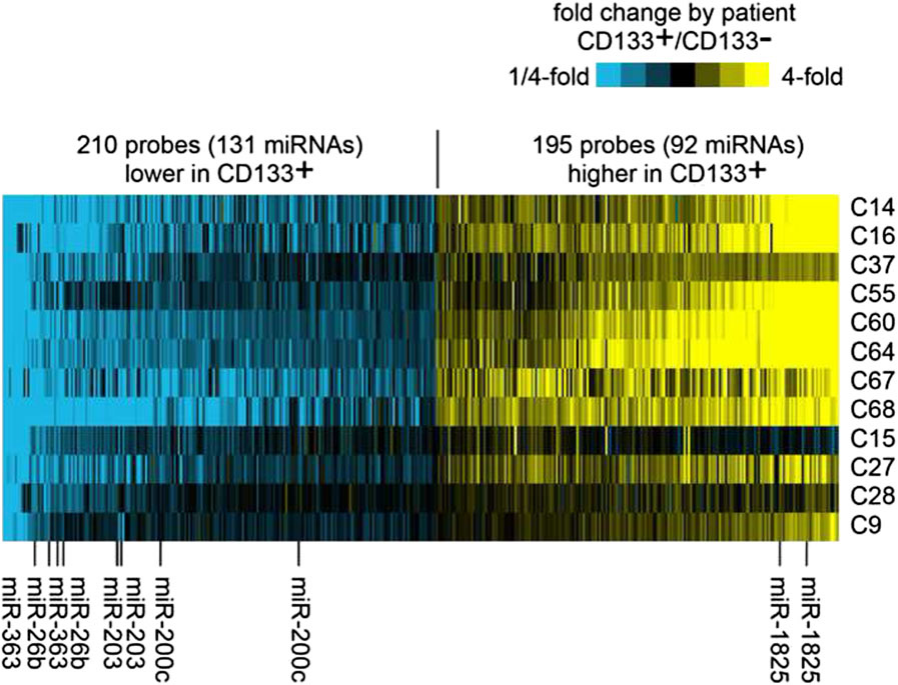
Heat-map representation of top differentially expressed miRNAs (*p* < 0.01, paired t-test, fold change > 1.4) in CD133^+^ larynx CSLCs vs. CD133^−^ larynx tumor cells, across twelve different patients. Yellow, high fold change in CD133^+^ patient sample as compared to its corresponding CD133^−^ paired sample; blue, high fold change in CD133^−^ sample compared to CD133^+^ paired sample

To analyze the global miRNA profile of CD133^+^ cells isolated from freshly resected LCa specimens, we performed microarray analysis using a discovery set comprised of 12 CD133^+^ and 12 CD133^−^ samples. Microarray profiling revealed that 405 probes were differentially expressed with a *p* value <0.01 (paired t-test) and with at least 1.4-fold change. A heat map representation of the deregulated miRNAs is shown in Fig. 1 (entire set of differentially expressed miRNAs are provided as a Additional file 1: Data Set). Among those significantly differentially expressed miRNAs, five downregulated (miR-26b-5p, miR-200c-3p, miR-203a, miR-223-3p, miR-363-3p) and three upregulated (miR-328, miR-574-3p, miR-1825) miRNAs were selected as a result of detailed literature search for further confirmation with qRT-PCR.

**Fig. 2:**
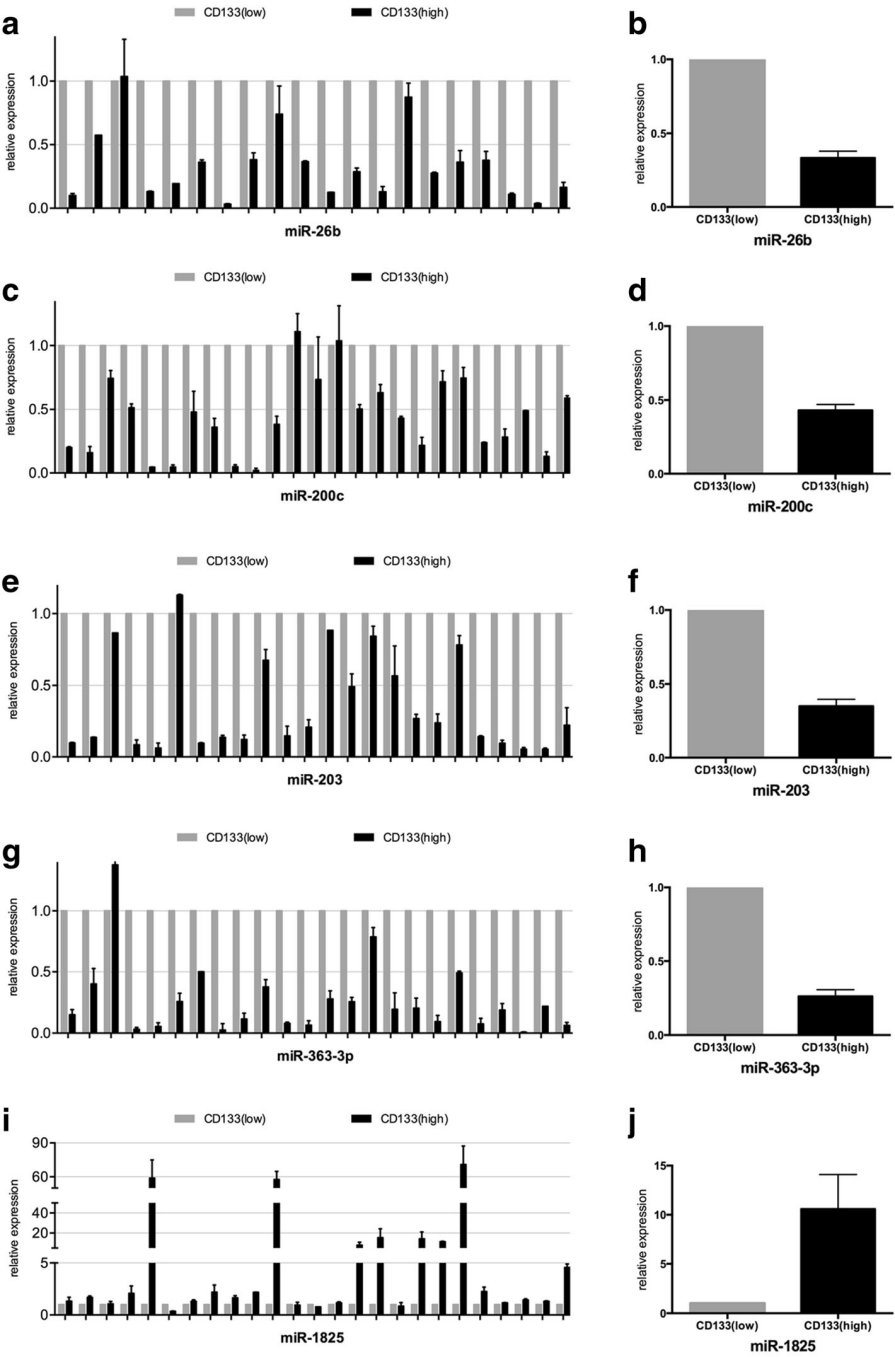
**a **Relative expression levels of miR-26b in each CD133^+^ and CD133^−^ sample pairs, and (**b**) mean relative expression levels miR-26b in CD133^+^ cells with respect to CD133^−^ cells. **c** Relative expression levels of miR-200c in each CD133^+^ and CD133^−^ sample pairs, and (**d**) mean relative expression levels miR-200c in CD133^+^ cells with respect to CD133^−^ cells. **e** Relative expression levels of miR-203 in each CD133^+^ and CD133^−^ sample pairs, and (**f**) mean relative expression levels miR-203 in CD133^+^ cells with respect to CD133^−^ cells. **g** Relative expression levels of miR-363-3p in each CD133^+^ and CD133^−^ sample pairs, and (**h**) mean relative expression levels miR-363-3p in CD133^+^ cells with respect to CD133^−^ cells. **i** Relative expression levels of miR-1825 in each CD133^+^ and CD133^−^ sample pairs, and **j** mean relative expression levels miR-1825 in CD133^+^ cells with respect to CD133^−^ cells

**Fig. 3:**
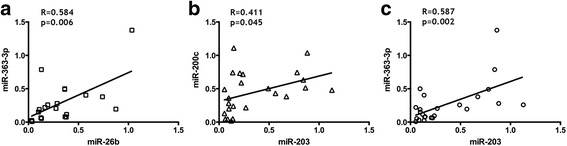
Scatter plot representation of (**a**) miR-26b/miR-363-3p, (**b**) miR-200c/miR-203, (**c**) miR-203/miR-363-3p expression correlation in CD133^+^ samples

The qRT-PCR results confirmed that five of the eight selected miRNAs had a differential expression between groups: miR-26b, miR-200c, miR-203, miR-363-3p, and miR-1825 (Fig. 2, *p* values and fold changes are provided in Table 2). Among those, miR-26b (Fig. 2a, b), miR-200c (Fig. 2c, d), miR-203 (Fig. 2e, f), and miR-363-3p (Fig. 2g, h) were found to have significantly reduced expression in CD133^+^ larynx CSLCs, whereas miR-1825 (Fig. 2i, j) were validated to have increased expression in these CD133 enriched LCa cells. However, expression levels of miR-223-3p, miR-328, and miR-574-3p were not significantly different between CD133^+^ vs. CD133^−^ LCa samples (Additional file 2: Figure S1, *p* values and fold changes are provided in Table 2). Although there was no statistically significant difference in the expression of miR-328 in CD133^+^ samples, its expression had a tendency to be elevated in CD133 enriched cell populations (Additional file 2: Figure S1C, D, Table 2). We further analyzed these miRNAs’ expressions with regard to T stage and histological stage of tumor samples. Results showed that miR-203 has lower expression in stage III samples compared to stage II tumor samples. Besides, miR-1825 has a tendency to have increased and miR-363-3p and miR-203 have a tendency to have decreased expression in T4 stage tumors compared to early stage tumors, although not significant. Since, miRNAs work in combination with each other rather than working individually and they operate in overlapping regulatory networks, demonstration of miRNAs’ correlation might be considered as indicative for their collaborative functioning in cells [34]. We, therefore, performed correlation analysis for the miRNAs found significantly deregulated between CD133^+^ and CD133^−^ cells. To evaluate their correlation, we used Pearson correlation analysis, which demonstrated that miR-26b, miR-200c, and miR-203 expressions were significantly correlated with miR-363-3p, miR-203, and miR-363-3p expressions, respectively, in CD133^+^ LCa tissue samples (Fig. 3).

**Table 2:** Fold Changes and *p* values for miRNAs evaluated with qRT-PCR

miRNA	Fold Change CD133^+^/CD133^−^	*p* value
miR-26b	0,333	**5,10007E-13**
miR-200c	0,434	**2,5502E-12**
miR-203	0,350	**1,71596E-12**
miR-223-3p	1,074	0,581
miR-328	1,950	0,268
miR-363-3p	0,263	**1,0634E-15**
miR-574-3p	0,894	0,599
miR-1825	10,583	**0,023**

*P* values lower than 0.05 are indicated as bold

### Relevant biological pathways affected from differentially expressed miRNAs

To explore the relevant biological pathways, which could be affected by the differential expression of miR-26b, miR-200c, miR-203, miR-363-3p, and miR-1825, we utilized DIANA miRPath v2.0, which revealed that several pathways overrepresented with a *p*-value <0.05, including cancer pathways (Table 3). Furthermore, miRWalk analysis showed that 3’UTR of several oncogenes were predicted to be targeted by miR-26b (202 out of 348 oncogenes), miR-200c (161 out of 348 oncogenes), miR-203 (216 out of 348 oncogenes), and miR-363-3p (175 out of 348 oncogenes). In addition, various tumor suppressors were estimated to be targeted by miR-1825 (29 out of 82 tumor suppressors). As to the analysis of validated targets of these miRNAs, miRTarBase database analysis revealed that miR-26b, miR-200c, miR-203, and miR-363-3p, and miR-1825 cooperatively target stem cell associated signaling pathways like Wnt, Hedgehog, and Notch (Fig. 4). Taken together, these analyses proposed that differential expression of miRNAs reported here might deregulate critical pathways involved in both carcinogenesis and establishment of CSLCs features.

**Table 3:** Overrepresented pathways, which could be affected by the differential expression of miR-26b, miR-200c, miR-203, miR-363-3p, and miR-1825

#	KEGG pathway	*p*-value	# genes	# miRNAs
1.	p53 signaling pathway	<0,001	17	3
2.	Viral carcinogenesis	<0,001	33	4
3.	Small cell lung cancer	0,002	17	2
4.	Chronic myeloid leukemia	0,003	3	2
5.	Pathways in cancer	0,005	6	3
6.	Hepatitis B	0,009	1	1
7.	Prostate cancer	0,018	3	2
8.	ECM-receptor interaction	0,019	1	1
9.	Glycosaminoglycan biosynthesis - chondroitin sulfate	0,023	5	1
10.	Glioma	0,026	2	2
11.	Cell cycle	0,028	3	2
12.	ErbB signaling pathway	0,043	2	2
13.	Epstein-Barr virus infection	0,044	29	2

**Fig. 4:**
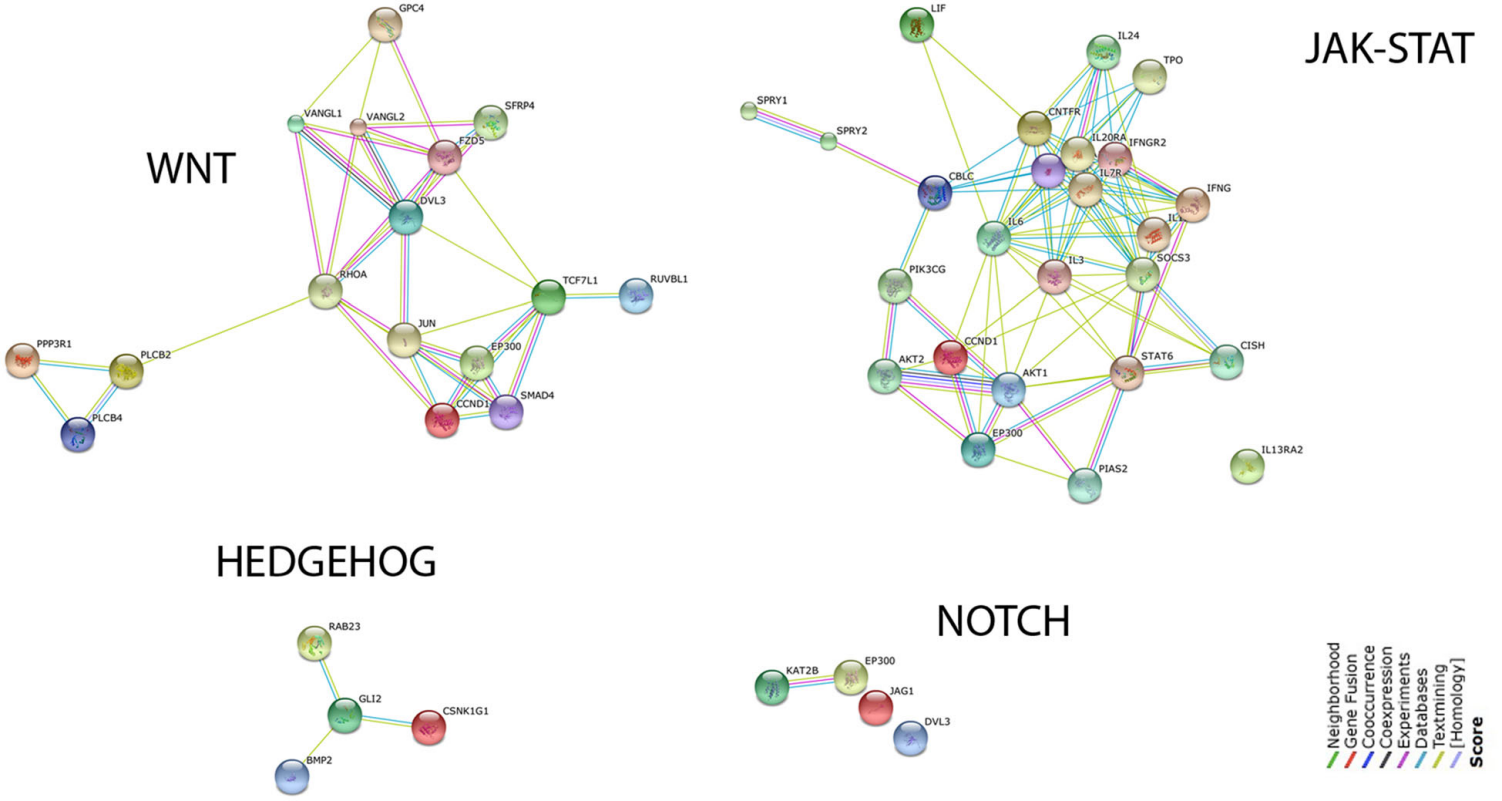
Validated targets of miR-26b, miR-200c, miR-203, and miR-363-3p, and miR-1825 are members of stem cell associated signaling pathways

## Discussion

MiRNAs have emerged as an abundant class of small RNAs implicated in post-transcriptional gene regulation. Since every single miRNA can potentially target hundreds of genes, their cooperative and additive regulation have been postulated to have profound impacts on multiple pathways simultaneously [35]. In addition to their extensively studied roles in tumor biology, they were also proposed to participate in establishing stem cell associated features [36]. MiRNA-driven pathways were demonstrated to be fundamental for oncogenesis as well as acquisition and maintenance of CSLCs characteristics [37, 38]. However, currently, little is known about the miRNA expression profiles of CSLCs. Therefore, there is a need for comprehensive characterization of the miRNAs that might be involved in the acquisition and maintenance of stemness properties of CSLCs.

In this study, we enriched for CSLCs using CD133 surface marker, which are previously demonstrated to have increased potential for self-renewal and multi-lineage differentiating potency in vivo [39] and display elevated levels of stemness factors in LCa specimens [14]. We further investigated the miRNA profiles in CD133^+^ CSLCs to find out differences in miRNA expression that could distinguish them from their more differentiated progenies. Our findings identified a set of miRNAs in these cells, which might present valuable information for a better understanding of the molecular basis of carcinogenesis and regulation of cancer stem cell features.

Of the miRNAs investigated here, miR-1825 resides within 20q11.21 chromosomal region, where has been reported to be a recurrent gain of function abnormality in human embryonic stem cells and induced pluripotent stem cells [40–42]. Nguyen et al. reported that human embryonic stem cells with 20q11.21 amplification displayed increased colony forming potential and decreased apoptosis [43]. Interestingly, 20q11.21 amplification in human embryonic stem cells resulted in acquisition of a gene-expression signature enriched for cancer-associated genes [44]. Recently, miR-1825 expression was reported to be elevated in majority of prostate cancer samples [45], and its expression was found to be upregulated in pancreatic cancer tissues in comparison to normal pancreatic duct [46]. In this study, we found overexpression of miR-1825 in CD133^+^ larynx CSLCs and suggest miR-1825 as an important contributor of carcinogenesis as a result of its dysregulation in CD133^+^ larynx CSLCs.

On the other hand, miR-363-3p, derived from the miR-106a-363 cluster on chromosome X, has been previously reported to be dysregulated in multiple cancers [47]. Although it acts different in distinct tumors, it has been demonstrated to behave like a tumor suppressor in several tumors such as colorectal cancer [47], nasal-type natural killer/T-cell lymphoma [48], head and neck squamous cell carcinoma [49], and hepatocellular carcinoma [50]. Interestingly, MYC, which has been implicated in stem cell self-renewal, maintenance of pluripotency, and control of cell fate decisions as well as carcinogenesis [51], was reported to directly bind to promoter of miR-363-3p and inhibit its expression [50]. MYC was also found to be destabilized by miR-363-3p through directly targeting and inhibiting USP28 [50] in hepatocellular carcinoma, pointing to a putative role for miR-363-3p in contribution to carcinogenesis and establishment of stemness features. Furthermore, miR-363-3p was found to directly target and repress GATA6, which is a transcription factor enhancing the expression of LGR5 in colorectal cancer [47]. LGR5, under the regulation of Wnt pathway, was proposed as a stem cell marker [52, 53] and its expression has been found to be overexpressed in various cancer tissues [54, 55]. These findings strengthen the potential role of miR-363-3p as a CSLCs specific miRNA in larynx pathogenesis. Additionally, miR-363 was reported to be repressed in head and neck squamous cell carcinoma tissues with lymph node metastasis and cell lines with increased invasive potential [49]. Ectopic expression of miR-363-3p decreased in vivo metastatic capacity of human neuroblastoma cells [56] and reversed the resistance of the breast cancer cell to the chemotherapeutic agent cisplatin [57]. Considering these findings and those of our own study, we suggest miR-363-3p as a strong candidate for establishment of stemness of CD133^high^ CSLCs.

MiR-26b expression has been found to be downregulated in tongue [58], nasopharyngeal carcinoma [59], and oral cancers [60]. Exposure to cigarette smoke, as a major risk factor for LCa, was proposed to cause repression of miR-26 family members’ expressions in animal models [61]. Besides, recent findings indicated epigenetic silencing of miR-26a/b in cancer cells particularly through aberrant DNA hypermethylation [62, 63]. MiR-26b also displayed low levels of expression in a human embryonic stem cell line (HUES-17) and in a colorectal cancer cell line with a high metastatic potential (LoVo) [64]. Loss of miR-26b also enhanced migration and invasion in oral squamous cell carcinoma [65]. Additionally, miR-26b expression in neural stem cells was found to be induced during their differentiation into neurons in vivo [66]. MiR-26b was also reported to be overexpressed during osteogenic differentiation of unrestricted somatic stem cells, which comprise a rare subpopulation in human cord blood [67]. We, in the present study, showed that CD133^+^ LCa cells possess reduced miR-26b expression. Given that miR-26b is downregulated in both stem cells and cancer cells, our findings suggest miR-26b as a CSLC specific miRNA, whose deregulation might participate in oncogenic transformation and maintenance of stem cell state in larynx CSLCs and as well as others.

MiR-200c and miR-203 have previously been extensively studied in various contexts, and both miRNAs are associated with the stemness of normal stem cells and CSLCs. Loss of miR-200 expression was observed during conversion of immortalized human mammary epithelial cells to a stem-like phenotype [68]. CSLCs isolated from metastatic breast tumor tissues also exhibited reduced miR-200 expression [68]. Besides, miR-200c was found to be strongly downregulated in Oct3/4, Sox2, and Nanog overexpressing CD133^+^ ovarian cells [69]. MiR-200c expression was significantly reduced in ALDH1+/CD44+ cells with cancer stem cell potency in head and neck squamous cell carcinoma [70]. Additionally, miR-200c was reported to repress epithelial-to-mesenchymal transition through directly targeting and inhibiting ZEB1 and ZEB2 transcription factors [71]. As one of the central regulators of epithelial mesenchymal transition (EMT), abnormal expression of miR-200c was found to alter several important biological processes implied in cell–cell contact, cell adhesion and motility [72]. Interestingly, reduced miR-200c expression was significantly correlated with recurrence in LCa [73].

As to miR-203, it has been found to be downregulated in head and neck region cancers including LCa [60, 74, 75]. MiR-203 was found to induce differentiation of normal epidermal stem cells [76, 77] and its expression was reported to be inhibited during EMT in stem cell-enriched cancer cell subpopulation [78]. Lower miR-203 expression was significantly associated with poor differentiation, advanced clinical stages, lymph node metastasis and decreased 5-year overall survival in LCa [79]. Additionally, miR-200c and miR-203 cooperatively inhibit stem cell factors’ expressions in both cancer cells and mouse embryonic stem cells [80]. In this study, we have found miR-200c and miR-203 to be downregulated in CD133^+^ larynx CSLCs, supporting their potential involvement in carcinogenesis as driving forces for tumor initiation, progression, metastasis, and recurrence. We here also demonstrated that miR-200c, and miR-203 expressions were significantly correlated with miR-203, and miR-363-3p expressions, respectively, in CD133^+^ LCa tissue samples. Correlation of those microRNAs expressions supports a recent report, which demonstrated that decreased expressions of miR-200c, miR-363, and miR-203 were associated with poor prognosis in human head and neck squamous cell carcinoma [81]. Besides, coordinated loss of miR-200c and miR-203 has been found to result in enhanced translation of the multiple targets and chronic activation of NF-κB, PI3K-Akt, and Ras-Erk pathways, leading to B cell transformation, which suggest that collaborative actions of multiple miRNAs rather than a single miRNA ensure the robustness of biological processes [82].

## Conclusion

In addition to miR-200c and miR-203, which have been demonstrated in distinct cancers as having CSLCs specific deregulation pattern, we propose miR-1825, miR-363-3p, and miR-26b as specific miRNAs with potential roles in acquisition and maintenance of stem cell associated features as well as in contributing to tumor initiation, progression, metastasis, chemoresistance, and recurrence. However, further detailed investigations are needed for each of the miRNAs studied here, to elucidate their roles in carcinogenesis and establishment of CSLCs related features.

## Additional files

Additional file 1: Data Set Entire set of top differentially expressed miRNAs is given in Additional Data Set. For each miRNA probe, fold change and *p* value information is provided. (XLSX 30 kb)
Additional file 2: Figure S1. (A) Relative expression levels of miR-223 in each CD133^+^ and CD133^−^ sample pairs, and (B) mean relative expression levels miR-223 in CD133^+^ cells with respect to CD133^−^ cells. (C) Relative expression levels of miR-328 in each CD133^+^ and CD133^−^ sample pairs, and (D) mean relative expression levels miR-328 in CD133^+^ cells with respect to CD133^−^ cells. (E) Relative expression levels of miR-574-3p in each CD133^+^ and CD133^−^ sample pairs, and (F) mean relative expression levels miR-574-3p in CD133^+^ cells with respect to CD133^−^ cells. (JPG 392 kb)

## Abbreviations

3′UTR: 3′-Untranslated regions
cDNA: First strand DNA
CSLCs: Cancer stem-like cells
LCa: Larynx cancer
MACS: Magnetic-activated cell sorting
miRNAs: MicroRNAs
mRNAs: Messenger RNAs
qRT-PCR: Quantitative reverse transcription PCR
