# Supplementary figures and images for: Identification of microRNA profile specific to cancer stem-like cells directly isolated from human larynx cancer specimens
Source: BMC Cancer. 2016 Nov 5;16:853. doi: 10.1186/s12885-016-2863-3 (PMC5097853; doi:10.1186/s12885-016-2863-3)

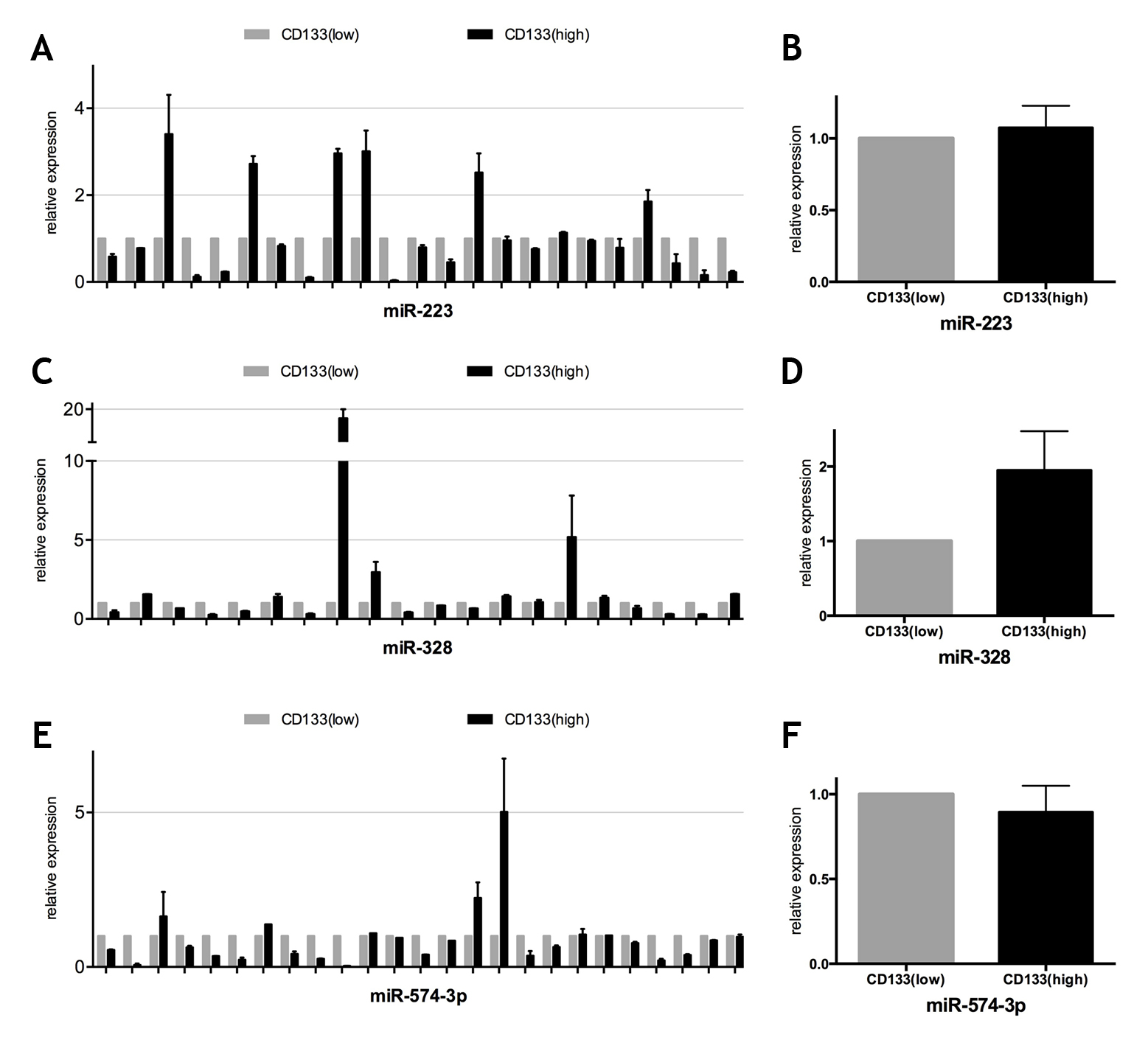

Supplement: Additional file 2: Figure S1. — (A) Relative expression levels of miR-223 in each CD133+ and CD133− sample pairs, and (B) mean relative expression levels miR-223 in CD133+ cells with respect to CD133− cells. (C) Relative expression levels of miR-328 in each CD133+ and CD133− sample pairs, and (D) mean relative expression levels miR-328 in CD133+ cells with respect to CD133− cells. (E) Relative expression levels of miR-574-3p in each CD133+ and CD133− sample pairs, and (F) mean relative expression levels miR-574-3p in CD133+ cells with respect to CD133− cells. (JPG 392 kb) [file 12885_2016_2863_MOESM2_ESM.jpg]
